# Microscale organization and separability of upper extremity representations in the human motor homunculus

**DOI:** 10.21203/rs.3.rs-9528027/v1

**Published:** 2026-04-29

**Authors:** Pedro G. L. B. Borges, Qasim Qureshi, Pierce Davis, Zhengjia Wang, Krishna Sargur, Jarl Haggerty, Katie Wingel, Min Jae Kim, Thomas J. Pisano, Elton Ho, Katrina Barth, Yoon Woo Byun, Joshua S. Miller, Jacqueline Dister, Ramin Anushiravani, Mark Murphy, Adam J. Poole, Jayme Strauss, Craig H. Mermel, Liming Qiu, Casey H. Halpern, Daniel Yoshor, Michael Beauchamp, Bijan Pesaran, Benjamin I. Rapoport, Iahn Cajigas

**Affiliations:** 1 Department of Neurosurgery, Perelman School of Medicine, University of Pennsylvania, Philadelphia, PA, USA; 2 Perelman School of Medicine, University of Pennsylvania, Philadelphia, PA, USA; 3 Department of Bioengineering, University of Pennsylvania, Philadelphia, PA, USA; 4 Penn Medical Scientist Training Program (MSTP), University of Pennsylvania, Philadelphia, PA, USA; 5 Department of Neurology, Perelman School of Medicine, University of Pennsylvania, Philadelphia, PA, USA; 6 Precision Neuroscience Corporation, New York, NY, USA.; 7 Department of Neuroscience, University of Pennsylvania, Philadelphia, PA, USA

**Keywords:** brain computer interfaces, neuroengineering, somatotopy, motor rehabilitation

## Abstract

Understanding the microscale spatial organization of the human motor homunculus is essential for designing surface-based brain-computer interfaces (BCIs). We investigated these dynamics using the highest-density clinically available subdural microelectrode arrays (1024 channels, 400 micrometers pitch) temporarily implanted in 11 neurosurgical patients undergoing awake surgery. We mapped broadband high gamma activity (>80 Hz) during upper extremity movements across 9 joints and hand gestures (rock, paper, scissors). Gestures produced consistent, localized spatial patterns in M1/S1, revealing shared microscale hand somatotopy across participants. Joint mapping revealed somatotopic representations organized as concentrically larger activation regions from distal to proximal joints. We characterized persistent spatial gradients in high gamma activity and representational overlap at microscale resolution. While previous macroscale studies showed overlapping motor representations, our high-density recordings provided a much finer mapping of this overlap and revealed a relationship between overlap degree and decoding performance. Our findings reveal a previously unobserved microscale mapping of motor commands in M1 and S1 and suggest that finer spatial resolution is necessary to decode complex movements from the brain surface.

## Introduction

The somatotopic organization of the brain refers to the principle that specific regions of the brain are associated with specific regions of the human body. This insight was first formalized by Penfield and Boldrey in 1937 by applying electrocortical stimulation (ECS) to the surface of the brain.^1^ Early incorporation of electrocorticography (ECoG) — electrodes on subdural or epidural spaces that do not penetrate the brain surface^2^ — showed that movement-evoked activity also demonstrates hand and finger somatotopy. This location specificity is particularly pronounced in the high gamma range (>60 Hz). High gamma activity (HGA) has been shown extensively to correlate with movement in ECoG literature and has been leveraged with ECoG-based brain computer interfaces (BCI) for motor rehabilitation.^3–6^

Clinical ECoG grids typically have coarse electrode spacing (pitch) of 1 – 10 mm.^7^ This spacing limits the resolution of somatotopic mapping. More recent work showed native representations of the upper extremity (UE)^8,9^ to be overlapping on the brain’s surface at those scales, even across the more spatially local high frequencies. Denser spatial sampling at sub-millimeter scales has been proposed as a potential improvement. To our knowledge, this approach has yet to be tested in humans.

We sought to answer the following questions: how is movement represented on the surface of the brain at microscale resolution? Does representational overlap persist at this scale, and how does it affect decoding? How does microscale organization compare between coordinated hand gestures and isolated joint movements? Are there any patterns across patients that may be used to guide future electrode placement?

Our study is, to our knowledge, the first characterization of native motor representation through high density microelectrode array (hdMEA). For this, we recorded from 11 temporarily-implanted patients undergoing awake surgery for DBS placement. Participants carried out a sequence coordinated hand gestures to test relationships between spatial extent, signal amplitude and discriminability between gestures. Extent of activation (EoA) predicted decoder performance, with extension movements recruiting larger cortical territories than flexion movements. Gestures recruited larger activation areas than isolated joint movements. Microscale resolution better delineates the extent of representational overlap across the arm, revealing hierarchical organization: proximal joints recruit larger territories that encompass nested distal representations. Cross-participant analysis identified consistent spatial gradients that may inform electrode placement strategies for brain-computer interfaces.

## Results

### Characterizing Neural Encoding Of Fine Motor Hand Gestures.

**Fig. 1.**
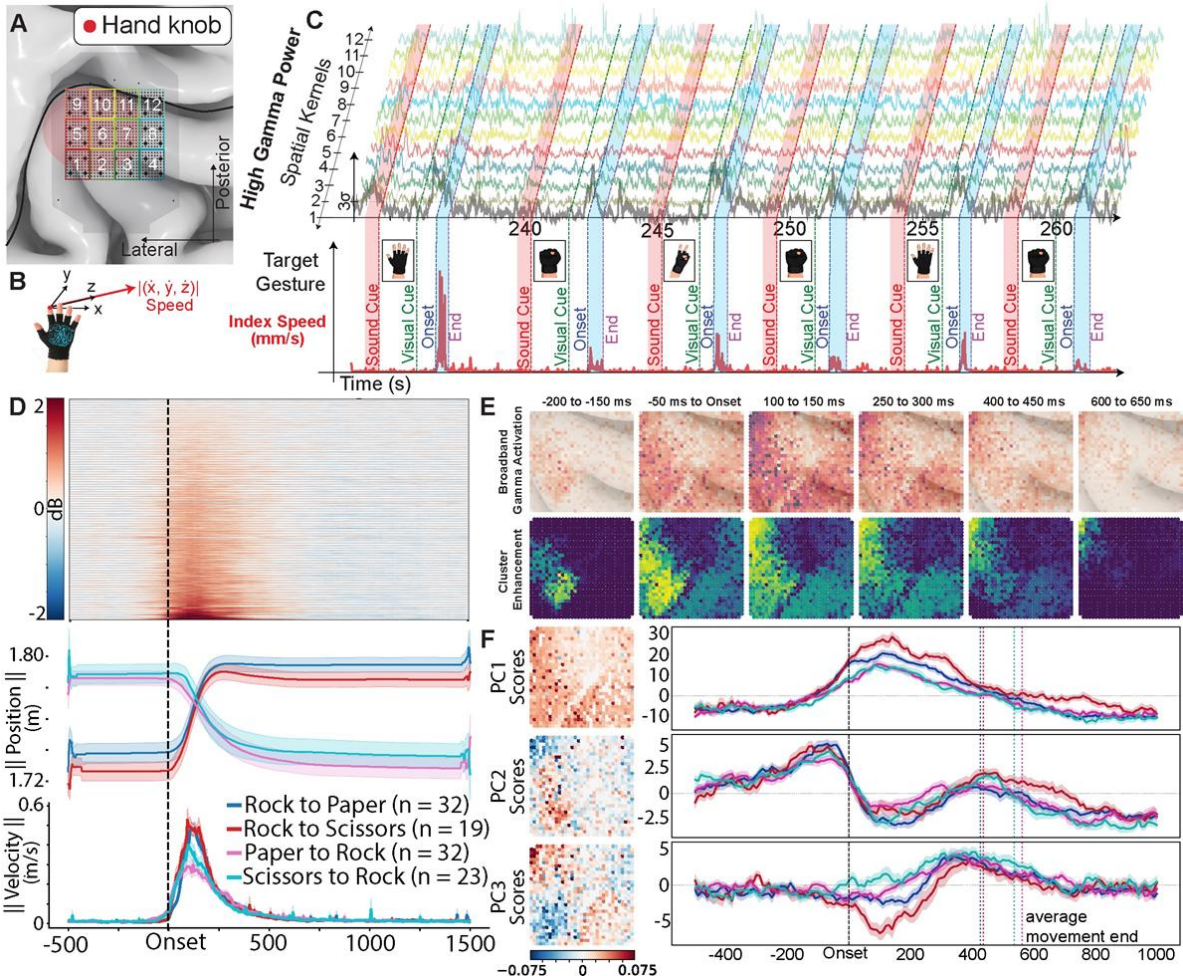
High-density electrocorticography reveals spatiotemporal dynamics of hand motor cortex during rock-paper-scissors gestures. **A.** Electrode array placement over the hand knob region of primary motor cortex for Participant 12. The array is overlaid on a 3D cortical reconstruction. Colored squares with numbered labels indicate 8×7 channel kernels used for spatial pooling to generate the high gamma traces shown in panel C. These were used solely for visualization purposes. Scale bar and anatomical orientation (posterior, lateral) are indicated. **B.** Motion capture system for tracking hand kinematics during gesture performance. Glove with stretch sensors (Stretch Sense) records 3D position data (ẋ, ẏ, ż). The magnitude of velocity from index and pinky finger sensors is used to segment movement trials. **C.** Representative neural and behavioral data across six trials of a single session for P12. Top: high gamma power traces from 12 spatially pooled channel kernels (colored by kernel identity) across multiple gesture transitions over a 30-second epoch. Bottom: index-finger speed profile with gesture-transition labels. Colored vertical lines mark task structure: sound cue (red), visual cue (green), movement onset (black), and movement end (purple). Red shaded regions highlight 500 ms baseline periods preceding each sound cue. Target gesture icons are displayed above each trial. **D.** Single-trial neural and kinematic data aligned to movement onset. Top: trial-averaged broadband high gamma power heatmap across all 1,024 channels (rows), showing dB change from baseline over time. The dashed vertical line indicates movement onset. Middle: Normalized position values for the index finger during each gesture transition trial. Bottom: index finger speed (m/s) aligned to the same time axis, illustrating the temporal coupling between neural activity and hand kinematics. **E.** Spatiotemporal evolution of cortical activation across six time epochs relative to movement onset, averaged across all gesture transitions. Top row: Broadband gamma activation showing dB change from baseline across the array. Each pixel represents one electrode, with warmer colors indicating greater activation. Bottom row: Threshold-free cluster enhancement (TFCE) scores computed using an 8-neighbor adjacency matrix to correct for family-wise error rates. Significant clusters emerge from baseline (−200 ms to −150 ms from onset) through peak activation (100–150 ms) and return to baseline by late epochs (600–650 ms). PrCS and CS denote precentral and central sulci, respectively. **F.** Principal component analysis (PCA) of high gamma population dynamics. PCA was performed on the channels × channels covariance matrix derived from the full channels × time data matrix. Left: spatial topographies of the first three principal components (PC1–PC3), with each pixel representing one electrode colored by its component loading. Right: corresponding time series of each component over the movement epoch. The three components capture distinct temporal phases of the motor response: PC1 reflects sustained broadband activation during movement execution; PC2 captures early pre-movement changes preceding motor onset, consistent with premotor anticipatory activity; and PC3 represents a late movement component emerging after peak activation. Together, these components provide a data-driven decomposition of the population-level spatiotemporal dynamics underlying gesture production.

Broadband high gamma activity (HGA) (80–200 Hz) was recorded from 1024 electrodes (1.2 × 1.2 cm area, 400 micrometers pitch) placed in M1 and surrounding regions as patients performed a rock-paper-scissors task with their hands during awake DBS surgery (Fig. 1A). In this task, trial starts were marked by a non-specific audio cue, and patients were instructed to follow along to a series of visual cues displayed on a monitor. We used motion capture to measure joint positions of major joints in the arm-hand during the task (Fig. 1B). Movement-related changes in HGA power were quantified in dB, using the ratio between power during movement and the mean baseline power across all trials (Fig. 1C). Baseline was defined as 500-millisecond period before each trial’s audio cue. Signed R^2^ metric was used to measure effect size, and extent of activation (EoA) was defined as sum of significant signed R^2^ values.^2,10–12^ Movement-related activity across patients was reported as dB changes on each individual trial or aggregate-trial signed R^2^ values from movement versus rest. This choice was made to ensure reported changes were not caused by outliers or sparse-trial high gamma increases. The signed R^2^ metric is the proportion of variance observed in the dataset that can be explained by movement and rest conditions, with a penalization term for unbalanced conditions. All analyses utilized Threshold-Free Cluster Enhancement (TFCE)^13^ to correct for family-wise error rates (FWER). Array locations were mapped to each participant’s native brains through RAVE.^14,15^ Clusters were defined as adjacencies of channels that showed similar movement-related evoked HGA.

### Spatiotemporal Dynamics Associated With Fine Motor Hand Representation.

Trial-averaged HGA rose above baseline 250 milliseconds before movement onset in M1 and returned to baseline by ~650 ms post-onset (Fig. 1D). To characterize these dynamics, we aligned trials to movement onset and captured significant HGA (p < 0.05, TFCE-corrected, two-tailed) from the earliest detectable deviation from baseline through movement completion. Movement-evoked potentials showed clusters in distinct cortical regions across participants: M1 (P5, P6, P9, P10, P12; n=5), S1 (P3, P10; n=2), MFG (P4; n=1), intersection between M1/SFG (P6; n=1). Participant 11 had a large central sulcus (CS) and was unable to be localized between M1 and S1. HGA and localization for each participant reported in **Supplemental Fig. 1**.

**Extended Data Figure 1.**
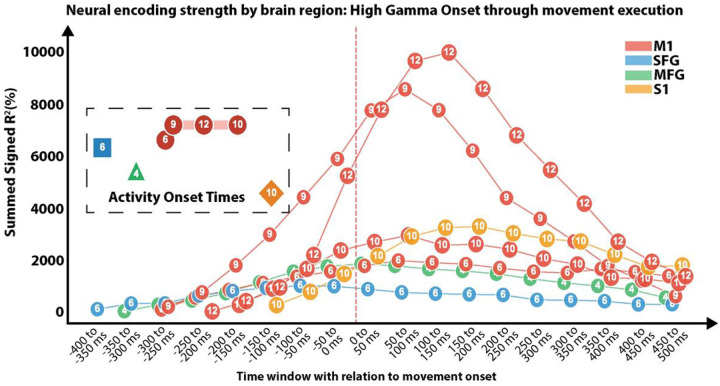
Temporal dynamics of neural encoding strength across cortical regions during gesture execution. Summed signed R^2^ (%) is plotted in 50-ms windows from −400 to +500 ms relative to movement onset (vertical red dashed line at 0 ms) for four cortical regions: primary motor cortex (M1, red; P6, P9, P10, P12), superior frontal gyrus (SFG, blue; P6), middle frontal gyrus (MFG, green; P4), and primary somatosensory cortex (S1, yellow; P10). Each marker shows the sum of signed R^2^ values across all electrodes within that region showing statistically significant modulation (TFCE-corrected, p < 0.025, two-tailed) for movement versus rest. Numbers inside markers indicate participant IDs; marker shape indicates region (square = SFG, triangle = MFG, circle = M1, diamond = S1). The dashed inset highlights activity onset times — defined as the earliest significant deviation from baseline — for each region: M1 onset −270 ± 45 ms (range −300 to −200 ms), S1 onset −175 ± 35 ms, MFG onset −350 ms, SFG onset −400 ms (M1 vs S1: Mann-Whitney U = 9.5, p = 0.105). M1 peaked at 35 ± 119 ms post-onset (peak latency 305 ± 110 ms, summed EoA = 48.4 ± 41.7), S1 later at 325 ± 212 ms (latency 500 ± 248 ms, EoA = 17.4 ± 21.9; U = 10.0, p = 0.071); SFG peaked pre-onset at −75 ms (EoA = 10.1), MFG at 25 ms (EoA = 18.5). Signed R^2^ is preserved with sign to indicate the direction of modulation in high gamma activity (80–200 Hz).

Mean and standard deviation for onset HGA modulation in M1 was −270.0 ± 44.7 ms pre-onset (range: −300 to −200 ms) and did not differ significantly to S1 (−175.0 ± 35.4 ms), likely reflecting the limited sample size (Mann-Whitney U = 9.5, p = 0.105). Clusters in MFG and SFG showed onsets of −350 ms and −400 ms, respectively. Peak activity timing varied by region: M1 peaked shortly after movement onset (35.0 ± 119.4 ms, latency: 305.0 ± 109.5 ms, EoA = 48.40 ± 41.74), while S1 peaked later (325.0 ± 212.1 ms, latency: 500.0 ± 247.5 ms, EoA = 17.43 ± 21.89; U = 10.0, p = 0.071). SFG peaked pre-onset (−75 ms, EoA = 10.09), MFG at 25 ms post-onset (EoA = 18.50). Activity for P11 was detectable at −150 ms, and peaked at 225 ms post-onset (EoA = 3.59) (Extended Data Fig. 1). These findings show movement-related HGA to precede the 150 ms starting time in previous studies with high density electrode arrays (pitch: 4 mm, 1.2 mm contact size).^9^

We identified strong correlations between HGA magnitude and spatial extent across all regions. In M1, correlations ranged from r = 0.550 to r = 0.966 across five arrays (P5: r = 0.550, p = 0.005; P6: r = 0.911, p < 0.001; P9: r = 0.944, p < 0.001; P10: r = 0.932, p < 0.001; P12: r = 0.966, p < 0.001). In S1, both arrays showed strong correlations (P3: r = 0.702, p < 0.001; P10: r = 0.901, p < 0.001). Single arrays in other regions also demonstrated robust relationships: MFG (P4: r = 0.879, p < 0.001) and SFG (P6: r = 0.807, p < 0.001). Greater amplitude HGA modulation is systematically associated with broader spatial recruitment on the surface of the brain at these scales. To our knowledge, this is the first formal observation of this relationship in humans.

### Distinct Surface Representations Of Hand Movement.

**Fig. 2.**
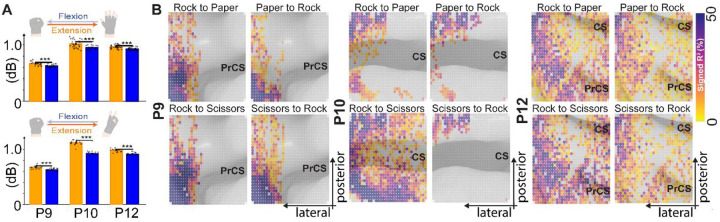
Asymmetric cortical representations of flexion versus extension movements revealed by high-density electrocorticography. **A.** Trial-averaged high gamma power (dB) comparing extension versus flexion movements across participants 9, 10 and 12. Top panel shows rock-paper transitions; bottom panel shows rock-scissors transitions. Each dot represents a single trial, with orange indicating extension movements (rock to paper/scissors) and blue indicating flexion movements (paper/scissors to rock). Bars indicate participant means. Extension movements showed significantly greater high gamma modulation than flexion movements across both gesture pairs (rock-paper: p = 0.002; rock-scissors: p < 0.001, independent samples t-test). Asterisks denote participants with statistically significant within-participant differences (*p < 0.05, ***p < 0.001). **B.** Spatial patterns of signed R^2^ values across the 1024-electrode array for three participants (P9, P10, P12) during all four gesture transitions. Each circle represents a single electrode, colored by signed R^2^ (percentage of variance in high gamma activity explained by movement versus rest). Extension movements (top row: rock to paper, rock to scissors) show greater spatial extent and magnitude of activation compared to flexion movements (bottom row: paper to rock, scissors to rock) across all participants. Flexion gestures exhibit more spatially similar activation patterns to each other than extension gestures, with this asymmetry consistently observed across participants. Arrows indicate anatomical orientation of the cortical surface. PrCS and CS denote precentral and central sulci, respectively.

We then sought to evaluate how movement-evoked potentials differed between gestures. Average HGA over time for each participant was significant (p-value < 0.05, TFCE-corrected, two-tailed) for 6 participants (P4, P6, P9, P10, P11 and P12). Of 11 enrolled participants, 8 showed significant HGA during gesture tasks (P3-P6, P9-P12), with P3 and P5 showing significant modulation only at individual timepoints but not in trial-averaged analyses. P11 was excluded due to the localization uncertainty and poor SNR secondary to sulcal anatomy. Therefore, subsequent analyses focus on the 5 participants with robust trial-averaged responses (P4, P6, P9, P10, P12). Within-participant mean HGA dB changes were larger for hand gestures involving finger extension (rock-to-paper and rock-to-scissors — see Methods section) in M1 and S1. This was also the case when pooling data across participants (rock-paper: extension 0.93 ± 0.13 dB vs. flexion 0.86 ± 0.16 dB, t = 3.17, p = 0.002; rock-scissors: extension 0.96 ± 0.21 dB vs. flexion 0.84 ± 0.16 dB, t = 3.92, p < 0.001) (Fig. 2A). The signed R^2^ related to High Gamma change for participants 9, 10 and 12 can be seen in Fig. 2B. While significant differences in high gamma power between gestures can be seen even when considering the array as a whole (Fig. 2A), spatial patterns (Fig. 2B) are more granular at the microscale. Flexural gestures (paper-to-rock, and scissors-to-rock) have HGA spatial patterns which are quantitatively more similar to each other than their extensor counterparts (rock-to-paper and rock-to-scissors).

### Surface Representation Of The Upper Extremity.

**Fig. 3.**
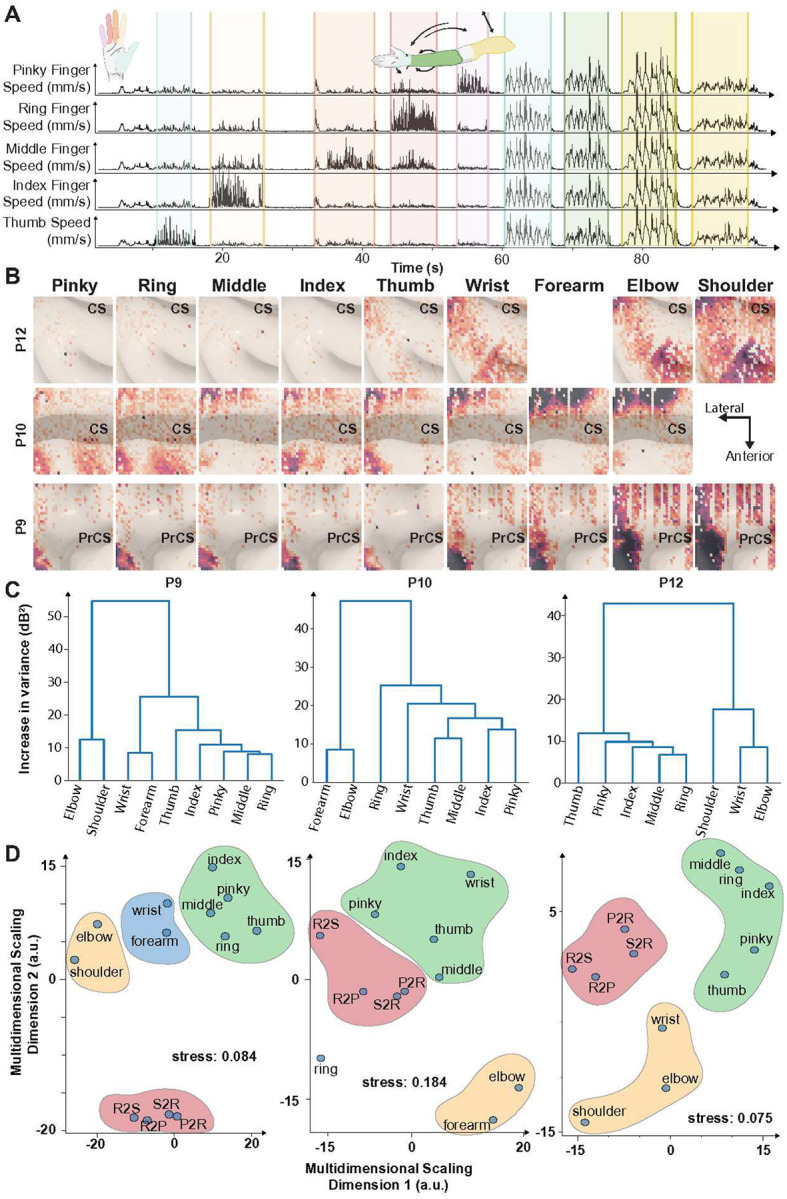
Somatotopic organization and representational similarity of upper extremity movements across the motor cortex. **A.** Task paradigm showing velocity tracings for upper extremity movements. The plot displays the maximum velocity magnitude across all motion capture sensors during the recording session, with colored regions indicating epochs for each body segment: individual fingers (thumb through pinky), wrist flexion/extension, forearm supination/pronation, elbow flexion/extension, and shoulder abduction/adduction. Hand schematic (left) illustrates the segmented body parts tracked during the task. Velocity peaks correspond to discrete movements of each joint, demonstrating the temporal structure of the motor task. **B.** Average-time dB change from baseline for three participants (P9, P10, P12) during movements of upper extremity joints. Each panel shows the average change in high gamma power (dB) from baseline during movement of the specified joint. Individual finger movements show more localized and weaker activation patterns compared to proximal joints. Wrist, forearm, elbow, and shoulder movements exhibit progressively larger spatial extent and magnitude of cortical activation, with distinct somatotopic organization visible across participants. CS denotes central sulcus; PrCS denotes precentral sulcus. **C.** Hierarchical clustering dendrograms for upper extremity joints across three participants. Dendrograms were generated using Ward linkage on Euclidean distances computed from high gamma activity patterns (dB change vectors) for each joint. Tree height represents the increase in within-cluster variance upon merging. Distinct clusters emerge separating distal joints (individual fingers) from proximal joints (shoulder, elbow, forearm), with wrist occupying an intermediate position. The clustering structure reveals consistent somatotopic organization of joint representations across participants. **D.** Multidimensional scaling (MDS) visualization of representational dissimilarity between upper extremity joints and hand gestures for each participant. Two-dimensional projections are derived from Euclidean distances between high gamma activity patterns. Joints are represented by labeled points; hand gestures (red points) are included for contextualization: R2P (rock to paper), R2S (rock to scissors), P2R (paper to rock), S2R (scissors to rock). Hand-drawn colored boundaries delineate clusters identified from hierarchical clustering. The spatial arrangement reveals a gradient from distal to proximal representations, consistent with somatotopic organization of the motor cortex. Normalized stress values indicate goodness of fit: P9 (stress = 0.084), P10 (stress = 0.184), P12 (stress = 0.075). MDS coordinates are in arbitrary units reflecting relative dissimilarity structure.

Characterization of UE movement on the surface of the brain is, to our knowledge, a significant gap in ECoG literature — especially in M1.^8^ We aimed to identify how spatially local this motor representation is, and how different joints presented at sub-millimeter inter-electrode scales. For this, we recorded from the hdMEA during a sequence of self-paced trials of isolated, periodic joint mobilizations (≥5 repetitions, ~10s each) (Fig. 3A). Rest periods were derived from no-movement intervals by looking at speed curves for each sensor of the UE (**Supplemental Fig. 2**). Global change from baseline (**Supplemental Fig. 3**), TFCE scores (**Supplemental Fig. 4**) and signed R^2^ metrics (**Supplemental Fig. 5**) were obtained following the same paradigm specified earlier (see Methods for more details).

We utilized Representational Similarity Analysis (RSA) to quantify discriminability between joints of the UE. Pairwise euclidean distances were computed between average HGA (dB change from rest, Fig. 3B) for each joint. The resulting similarity matrix was used to construct a dendrogram (Fig. 3C). Surface representations across the surface of M1 (P9 and P12) and S1 (P10) appear to follow clear hierarchical representation. Distances were projected to two dimensions through multidimensional scaling (Fig. 3C). Observed clusters align with anatomical distribution across the UE across the proximal-distal axis. Joints mobilized through actuation of forearm musculature were closer together (e.g., individual fingers), with varying distances to wrist, forearm and elbow, and shoulder abduction-adduction being the most dissimilar.

### Relationship Between Encoding Amplitude and Spatial Extent.

**Extended Data Figure 2.**
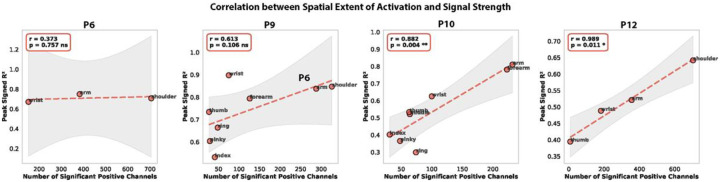
Per-participant correlation between cortical activation extent and signal magnitude during isolated joint movements. Each panel plots peak signed R^2^ (y-axis) against the number of significant positive channels (x-axis) for individual joints (thumb, index, middle, ring, pinky, wrist, forearm, arm, shoulder) in one participant. Dashed lines show ordinary least-squares regression fits with shaded 95% confidence bands. Significance threshold for inclusion in the channel count: signed R^2^ > 0, p < 0.05 (TFCE-corrected). Strong positive correlations were observed in P10 (r = 0.882, p = 0.004) and P12 (r = 0.989, p = 0.011); a strong but non-significant trend in P9 (r = 0.613, p = 0.106); no relationship in P6 (r = 0.373, p = 0.757). The pattern indicates that joints recruiting larger cortical territories also tend to produce stronger per-electrode modulation, mirroring the gesture-task relationship reported in Extended Data Fig. 4.

We sought to evaluate whether the same relationship between effect size (maximum signed R^2^) and spatial extent (number of significant channels) held across different joints. Correlations between these measures revealed some inter-subject variability. Very strong positive relationships were observed in P10 (r = 0.882, p = 0.004) and P12 (r = 0.989, p = 0.011), indicating that the relationship between strength of HGA relates to its spatial extent. A strong but not significant effect was also observed for P9 (r = 0.613, p = 0.106). No relationship was observed for P6 (r = 0.373, p = 0.757). (Extended Data Fig. 2)

### Surface Overlap In Upper Extremity Representations.

**Fig. 4.**
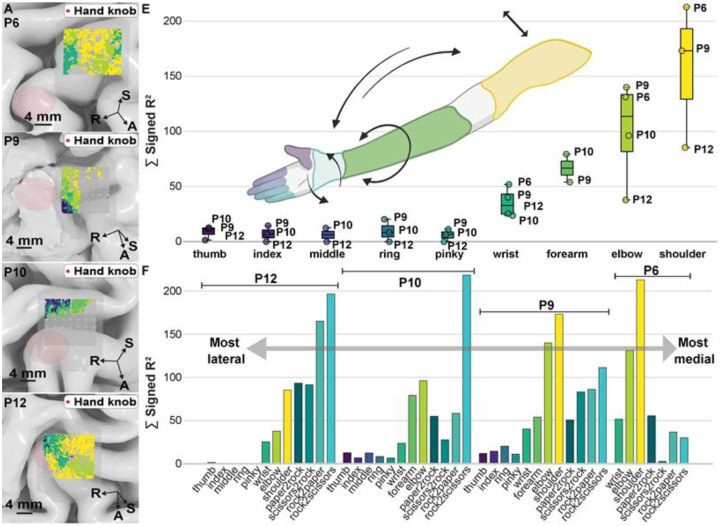
Spatial organization and cortical activation extent of upper extremity movements reveal somatotopic gradients and overlapping motor representations. **A-D.** Electrode array placement and activation patterns for participants P6, P9, P10, and P12. Each panel shows a 3D reconstruction of the cortical surface with the array positioned over the hand knob region of M1. Colored overlays indicate electrodes with significant activation (signed R^2^ > 0, p < 0.05) during upper extremity movements. Due to extensive overlap between joint representations, electrodes are colored according to the joint with the fewest active electrodes, illustrating the overlapping nature of motor representations across the array. Central sulcus is labeled for anatomical reference. Scale bar = 4 mm. **E.** Quantification of cortical activation extent across nine upper extremity joints for all participants. Box plots show the distribution of EoA (summed signed R^2^) values across participants, calculated by summing positive signed R^2^ values from all significantly activated electrodes for each joint movement. Individual data points represent individual participants (P6, P9, P10, P12), color-coded by participant identity. Proximal joints (shoulder, arm, forearm) recruit substantially larger cortical areas compared to distal joints (individual fingers), with shoulder movements showing the greatest activation extent. Wrist occupies an intermediate position between finger and proximal joint representations. Box bounds indicate 25th and 75th percentiles, center line shows median, and whiskers extend to data range. **F.** Spatial distribution of joint-specific activation along the mediolateral axis of the electrode array. Bars represent EoA (summed signed R^2^) values for each joint within spatial bins across the array, organized by participant (P12, P10, P9, P6) from left to right. Bar colors correspond to individual joints. A mediolateral rank gradient is apparent: proximal joint activations (shoulder, arm, wrist) show greater magnitude in medial regions and decrease laterally, while gesture-related activations (rock-paper-scissors movements) show the opposite pattern, increasing from medial to lateral. This complementary spatial organization further suggests functional segregation of proximal versus distal motor control along the mediolateral axis of primary motor cortex, though individual finger movements show insufficient data for clear gradient determination.

Changes in the recorded broadband HGA during movement recruited larger areas of M1 (P6, P9, P12) and S1 (P10) as larger and increasingly proximal joints were mobilized. Note that while P10 had electrodes spanning both M1 and S1, only S1 channels showed significant modulation during isolated joint movements, whereas both regions were significantly active during the rock-paper-scissors (RPS) gesture task. Average HGA formed patterns of nested, overlapping shapes in both M1 (P6, P9, P10, P12) and S1 (P10). The overlapping nature of these joint representations can be observed in Fig. 4A, in which the masked areas for significant channels were overlaid on top of the native brain representation. Due to extensive overlap between joint-related HGA, electrodes are colored according to the joint with the fewest active electrodes, illustrating the overlapping nature of motor representations across the array.

We observed that EoA increased as we moved towards more proximal joints of the contralateral upper limb. These gradients persisted across participants and can be shown plotted in Fig. 4B. Hierarchy between activation profiles obeyed a shoulder > elbow > forearm > wrist > individual digits both across M1 (participants 6, 9 and 12) and S1 (participant 10). Notably, when evaluating across the medial-lateral axis of the brain, EoA for shoulder, arm, and wrist decreased as we move laterally towards the hand knob. Hand gestures (represented in cyan-teal colors in Fig. 4C) followed the inverse relationship, with EoA increasing as we moved laterally.

**Fig. 5.**
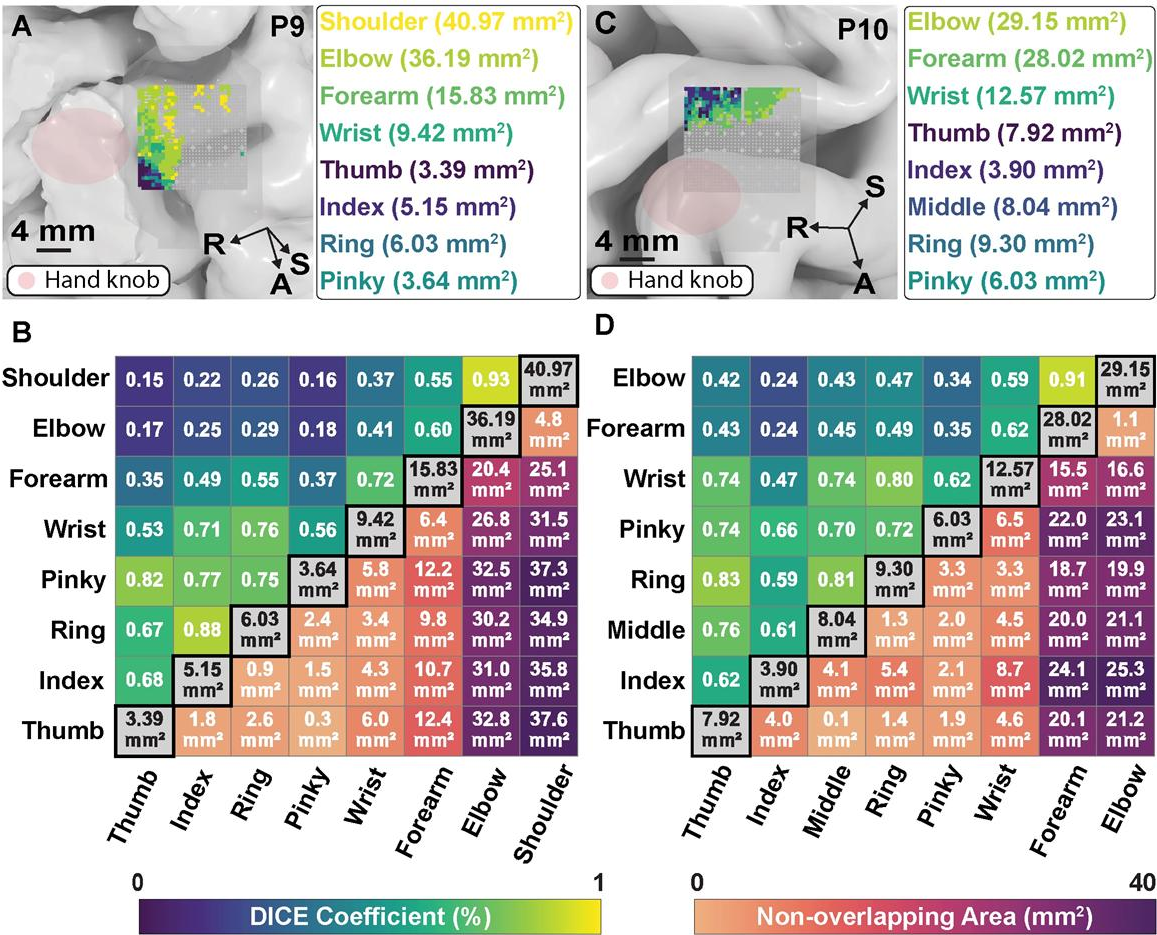
Quantification of cortical activation area and spatial overlap reveals hierarchical and overlapping motor representations across primary motor and somatosensory cortices. **A.** Thresholded activation map for P10 (S1) displaying similar somatotopic organization and activation patterns. Despite containing only S1 electrodes, P10 shows comparable cortical area allocation and spatial organization to P9 (**Panel A**), with proximal joints occupying larger territories (elbow: 29.15 mm^2^, forearm: 28.02 mm^2^) than distal joints (index: 3.90 mm^2^, pinky: 6.03 mm^2^). **B.** Pairwise spatial overlap analysis for P9 (M1) quantifying the relationship between joint representations. Upper diagonal shows Dice coefficients (0–1 scale, green to yellow) measuring spatial overlap between joint pairs, where 0 indicates no overlap and 1 indicates complete overlap. Diagonal (white/gray cells) shows total activation area for each joint in mm^2^. Lower diagonal (orange to purple) shows non-overlapping area between joint pairs in mm^2^, representing the exclusive cortical territory unique to each joint comparison. High Dice coefficients between adjacent joints (e.g., shoulder-elbow: 0.93) indicate substantial representational overlap, while more distant joints show progressively less overlap. **C.** Thresholded activation map for P10 (S1) displaying similar somatotopic organization and activation patterns. Despite containing only S1 electrodes, P10 shows comparable cortical area allocation and spatial organization to P9 (**Panel A**), with proximal joints occupying larger territories (elbow: 29.15 mm^2^, forearm: 28.02 mm^2^) than distal joints (index: 3.90 mm^2^, pinky: 6.03 mm^2^). **D.** Pairwise spatial overlap analysis for P10 (S1). Matrix organization identical to panel B. Despite having only S1 coverage, P10 demonstrates remarkably similar overlap patterns and Dice coefficients to P9, with high overlap between proximal joints (forearm-elbow: 0.91) and progressive decrease in overlap with increasing anatomical distance between joints. The consistency of these patterns across participants supports a conserved organizational principle of overlapping, hierarchical motor representations in primary motor cortex, with representation size scaling inversely with distance along the proximal-distal axis.

We further characterized the spatial extent of motor representation for these joints by quantifying the size and overlap of cortical representation areas for each movement category (Fig. 5). An area of π × (0.2 mm)^2^ ≈ 0.126 mm^2^ was assigned to each electrode with signed R^2^ > 0, p < 0.05. In P9 (M1), individual finger representations ranged from 3.39 mm^2^ (thumb) to 6.03 mm^2^ (ring), with representational area increasing systematically along the proximal-distal axis. The largest representations were attributable to proximal joints, with shoulder and elbow activating 40.97 mm^2^ and 36.19 mm^2^ respectively. Finger representations demonstrated high pairwise Dice coefficients, with notable overlap between adjacent digits (e.g., ring-index: 0.88). Wrist and forearm showed substantial overlap (Dice coefficient 0.72), while proximal joints like shoulder and elbow exhibited the highest overlap (0.93). In P10 (S1), the largest representations were for elbow and forearm, activating 29.15 mm^2^ and 28.02 mm^2^ respectively. Wrist and forearm showed substantial overlap (Dice coefficient 0.62), while proximal joints exhibited high overlap (0.91). Joint representations showed consistent organizational principles whether in M1 or S1 (represented by P9 and P10, respectively): considerable overlap between representations within each limb segment (arm-forearm-hand) and non-overlapping areas that progressively increased with anatomical distance between effectors, ranging from sub-millimeter differences between adjacent fingers to over 20 mm^2^ between distant joints.

### Extent Of Activation Predicts Decoding Performance Across The Movement Epoch.

**Extended Data Figure 3.**
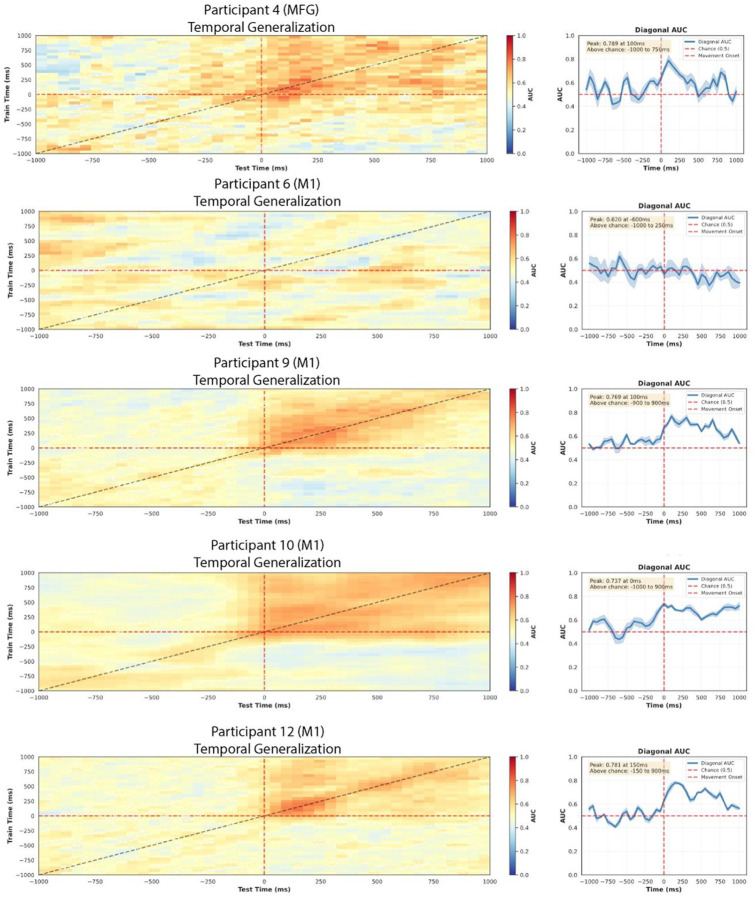
Temporal generalization of gesture-decoding classifiers in five participants. Rows show participants P4 (MFG), P6 (M1), P9 (M1), P10 (M1) and P12 (M1). Left column: temporal generalization matrices in which a one-versus-rest logistic regression classifier was trained at each 100-ms window (y-axis: train time relative to movement onset) and evaluated at every other window (x-axis: test time). Cell color encodes macro-averaged area under the receiver operating characteristic curve (AUC) for gesture classification (rock-to-paper, rock-to-scissors, paper-to-rock, scissors-to-rock; AUC chance = 0.5, 5-fold stratified cross-validation, L1-regularized logistic regression with C = 0.1). Diagonal cells reflect within-window decoding; off-diagonal extension reflects representational stability of the underlying neural code across time. Right column: diagonal AUC trace from each matrix plotted against time relative to movement onset (vertical red dashed line at 0 ms). Solid blue line shows the mean across folds, shaded region the standard error of the mean.

**Extended Data Figure 4.**
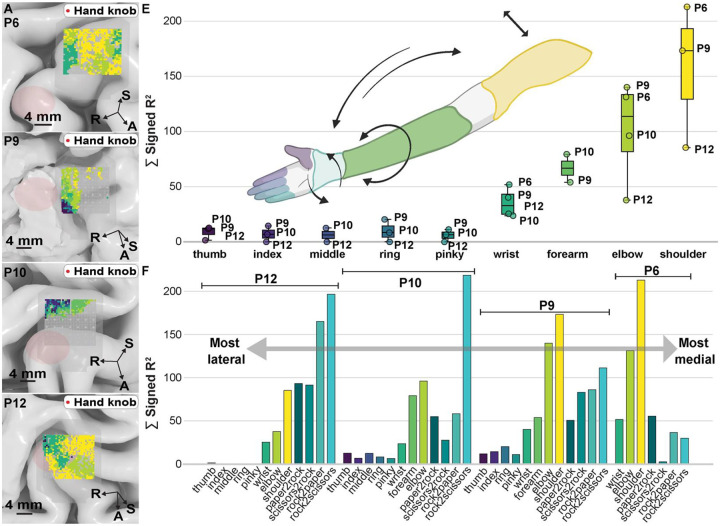
Per-participant-region correlation between cortical activation extent and signal magnitude during the rock-paper-scissors task. Panels show maximum signed R^2^ (y-axis, %) plotted against the number of significant channels (x-axis) across time bins spanning the movement epoch for each participant-region pair: P3 (S1), P4 (MFG), P5 (M1), P6 (M1), P6 (SFG), P9 (M1), P10 (S1), P10 (M1), and P12 (M1). Each dot represents one 50-ms time bin within the movement epoch; solid lines are ordinary least-squares regression fits. All participant-region pairs showed statistically significant positive correlations: P3 r = 0.702, P4 r = 0.879, P5 r = 0.550 (p < 0.01), P6 (M1) r = 0.911, P6 (SFG) r = 0.807, P9 r = 0.944, P10 (S1) r = 0.901, P10 (M1) r = 0.932, P12 r = 0.966 (all other p < 0.001). Pooled within M1 arrays, mean r = 0.910; pooled within S1 arrays, mean r = 0.826. The relationship quantifies how broader cortical recruitment systematically tracks larger per-electrode effect size during gesture production.

**Fig. 6.**
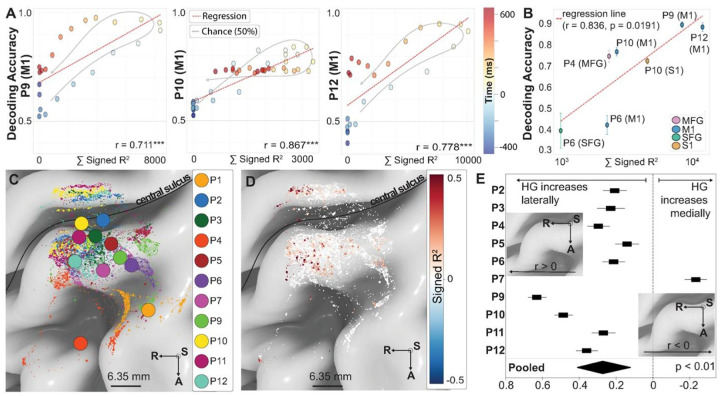
Spatial gradient of neural encoding strength reveals optimal electrode placement for hand gesture decoding in primary motor cortex. **A.** Temporal evolution of the relationship between neural encoding strength and decoding performance for three participants with electrodes in M1 (P9, P10, P12). Each scatter plot shows summed signed R^2^ values (x-axis) versus decoding accuracy (y-axis) at different time points from −400 to 650 ms relative to movement onset. Points are colored by time (blue = pre-movement, red = post-movement). Strong positive correlations were observed for all participants (P9: r = 0.711, P10: r = 0.867, P12: r = 0.778; all p < 0.001), demonstrating that moment-to-moment changes in high gamma modulation predict real-time decoding performance throughout the movement epoch. **B.** Relationship between cortical EoA and binary gesture decoding accuracy (hand open vs. closed) across participants and cortical regions. Each point represents a participant-region pair, colored by anatomical location: M1 (blue), MFG (pink), SFG (teal), and S1 (orange). The x-axis shows EoA (summed signed R^2^) in % (log scale) calculated across all statistically significant electrodes. The y-axis shows logistic regression decoding accuracy from 100 ms time windows. A significant positive correlation was observed (r = 0.836, p = 0.019, n = 7), indicating that greater cortical activation directly predicts improved gesture discrimination. Error bars represent ±1 SEM across cross-validation folds. Red dashed line shows the regression fit. **C.** Electrode array placements across all participants projected onto a standardized brain surface (fsaverage). Each colored dot represents an individual electrode, color-coded by participant (n = 11 participants: P1, P2, P3, P4, P5, P6, P7, P9, P10, P11, P12; 11,264 electrodes total). Large colored circles indicate the centroid of each participant’s array placement. Black outline delineates the central sulcus. Electrodes are distributed across the motor hand area with substantial inter-subject variability in placement. Orientation axes indicate posterior (P) and anterior (A) directions. Scale bar = 6.35 mm. **D.** Spatial distribution of neural encoding strength across the sampled motor cortex. Each dot represents an individual electrode, colored according to its signed R^2^ value (high gamma activity during movement versus rest), mapped to a divergent blue-white-red palette. Red indicates increased activation (positive R^2^), blue indicates suppression relative to rest (negative R^2^), and white indicates intermediate values. The spatial pattern reveals a medial-to-lateral gradient of increasing high gamma modulation, with the strongest responses concentrated in the lateral portion of the motor hand area near the hand knob region. Data aggregated across all participants (except P1, see Methods) after projection to common anatomical space (MNI-152). Scale bar = 6.35 mm. **E.** Within-participant correlation analysis confirming a significant medial-to-lateral gradient in neural encoding strength. Each row represents an individual participant, with squares indicating participant-specific correlation coefficients between electrode position along the medial-lateral axis (MNI-152 space) and high gamma activation (dB). Horizontal lines represent 95% confidence intervals. Participants are grouped by gradient direction: those showing higher gamma laterally (r > 0; P2, P3, P4, P5, P6, P7) and those showing higher gamma medially (r < 0; P9, P10, P11, P12). The bottom row shows the pooled effect across all participants using Fisher z-transformation (p < 0.01), demonstrating that electrodes placed more laterally within the motor hand area consistently show stronger task-related high gamma modulation.

We next asked whether the extent of activation measured at each moment in time tracked moment-to-moment decoding accuracy. For channels in M1, decoding performance was strongly correlated with the EoA throughout the movement epoch (−450 to 650 ms; P9: r = 0.711, p < 0.001; P10: r = 0.867, p < 0.001; P12: r = 0.778, p < 0.001). This result (Fig. 6A) demonstrates that greater high gamma modulation during movement directly predicted improved gesture discrimination. MVPA generalization matrices and temporal profiles for P4, P6, P9, P10 and P12 can be found on Extended Data Fig. 3. EoA integrates response magnitude and spatial extent which are highly correlated across motor cortex (pooled M1 r = 0.910; pooled S1 r = 0.826; Extended Data Fig. 4). This coupling strengthens our previous observation that stronger neural responses recruit broader cortical territories on the cortical surface. Partial correlations controlling for either component eliminated the EoA-decoding relationship, as expected when separating coupled phenomena. EoA’s predictive value comes from quantifying total cortical engagement rather than either component alone.

### Effect Of Surface Representation on Decoding Hand Open *versus* Closed.

We then used a binary classification algorithm to investigate the relationship between HGA and dexterous hand movement. For this, a logistic regression was trained on a sequence of 100-millisecond windows in a Multivariate Pattern Analysis approach. EoA was significantly correlated to mean decoding accuracy across folds (r = 0.836, p = 0.019, n = 7 participant-region pairs) (Fig. 6B). Decoding performance was above chance for M1 (74.7 ± 22.1%, n = 4), MFG (P4: 75.1%) and S1 (P10: 72.8%, n = 1). Decoding from SFG was not achieved (P6: 40.0%, n = 1).

### Effect Of Surface Representation on Decoding RPS.

**Extended Data Figure 5.**
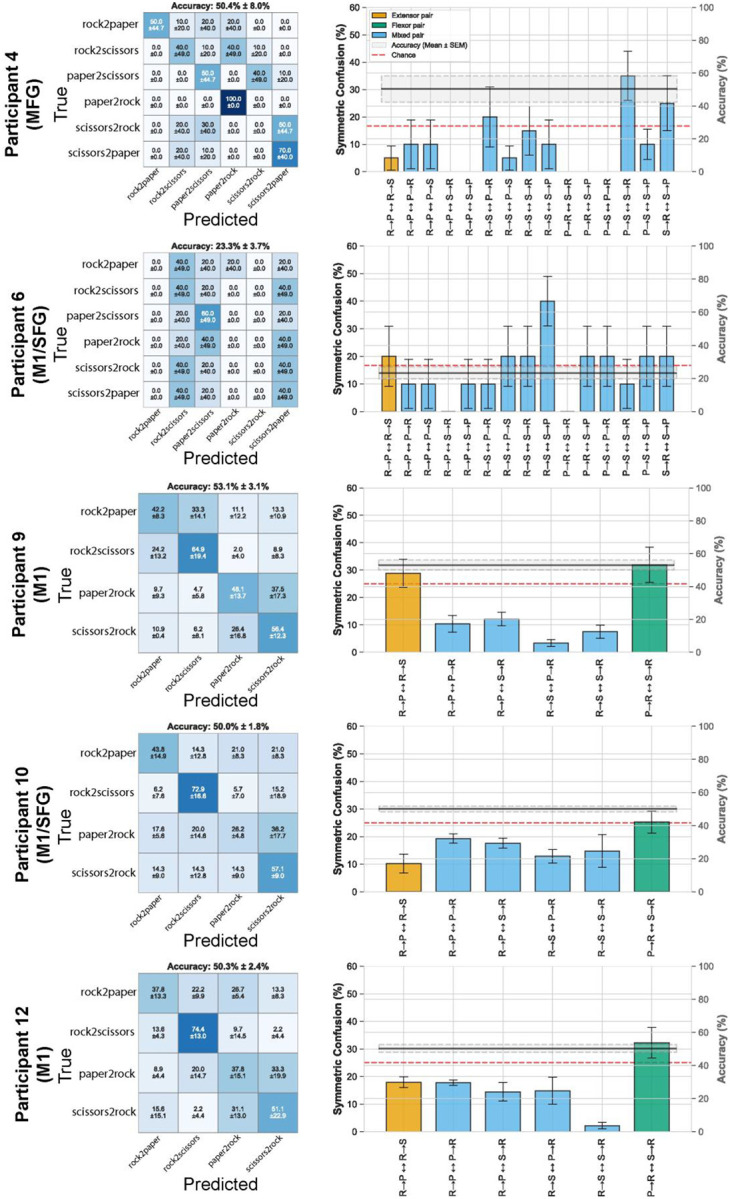
Confusion matrices and symmetric confusion rates gesture classification. Each row corresponds to one participant (P4 MFG; P6 M1/SFG; P9 M1; P10 M1/SFG; P12 M1). **A.** Normalized confusion matrices (left column) for one-versus-rest logistic regression on rock-paper-scissors transitions (rock-to-paper, rock-to-scissors, paper-to-rock, scissors-to-rock), evaluated with 5-fold stratified cross-validation. Cell color encodes the mean proportion of trials with the given true class (rows) classified as each predicted class (columns); diagonal cells indicate correct classifications. Mean accuracy ± SEM across folds: P4 = 50.4 ± 8.0%, P6 = 23.3 ± 3.7% (six-way; chance 16.67%); P9 = 53.1 ± 3.1%, P10 = 50.0 ± 1.8%, P12 = 50.3 ± 2.4% (four-way; chance 25%). **B.** Pairwise symmetric confusion rates (right column), computed for each gesture pair as the average of off-diagonal entries (i,j) and (j,i) from the confusion matrix in panel A. Bars are color-coded by gesture relationship: extension–extension pairs (orange), flexion–flexion pairs (blue), mixed extension/flexion pairs (green). Per-pair binary accuracy is overlaid on the right-hand y-axis (mean ± SEM across folds). Red dashed line marks chance for the binary comparison (50%). Across participants, flexion-dominated pairs (paper-to-rock vs. scissors-to-rock) showed systematically higher symmetric confusion than extension pairs (rock-to-paper vs. rock-to-scissors), consistent with greater spatial similarity between flexion-related cortical activation patterns reported **in Fig. 2B**.

Gesture classification exceeded chance in all five participants (5-fold CV; six-way [chance 16.67%]: P4 = 50.4 ± 8.0%, P6 = 23.3 ± 3.7%; four-way [chance 25%]: P9 = 53.1 ± 3.1%, P10 = 50.0 ± 1.8%, P12 = 50.3 ± 2.4%). Flexion-dominated pairs showed higher confusion than extension pairs (Extended Data Fig. 5). This pattern indicates that gestures sharing similar kinematic and spatial HGA profiles were systematically more difficult to distinguish than movements with distinct directional components.

### Spatial Gradients In Motor Representation Persist Across Array Placements.

Having shown that EoA predicts decoding accuracy within individual participants (Fig. 6A & B), we next asked whether EoA’s spatial organization was consistent enough across participants to inform electrode-placement strategies for future arrays. Within individual participants, we had observed systematic gradients in joint representations along the medial-lateral axis of the sampled surface: proximal-joint EoA increased medially, and hand-gesture EoA increased laterally toward the hand knob (see Fig. 4F). To test whether gesture representation was generalizable, each participant’s electrode coordinates were projected onto a normative brain (“fsaverage”) (Fig. 6C & 6D). Aggregate cortical coverage across participants is shown in **Supplemental Fig. 6**.

Within-participant correlations between MNI-152 coordinates and HGA are shown in Fig. 6E. Because the precentral gyrus curves inferiorly as it extends laterally, superior coordinates within our sampled region correspond approximately to more medial positions; the inferior-superior and medial-lateral gradients therefore partly reflect the same geometric axis rather than two independent organizational principles. A significant spatial gradient existed along the medial-lateral axis (mean r = 0.273, 95% CI: 0.121 to 0.412; t(9) = 3.47, p = 0.028, FDR-corrected), with high gamma activity increasing laterally toward the hand knob region. Consistent with the gyral geometry, a significant negative correlation emerged along the inferior-superior axis (mean r = −0.197, 95% CI: −0.323 to - 0.065; t(9) = −2.91, p = 0.035, FDR-corrected), indicating decreasing HGA with superior displacement. No significant gradient existed along the anterior-posterior axis (mean r = 0.064, 95% CI: −0.065 to 0.190; t(9) = 0.97, p = 0.357, FDR-corrected). P-values were corrected for multiple comparisons across three spatial axes using the Benjamini-Hochberg false discovery rate procedure.

**Extended Data Figure 6.**
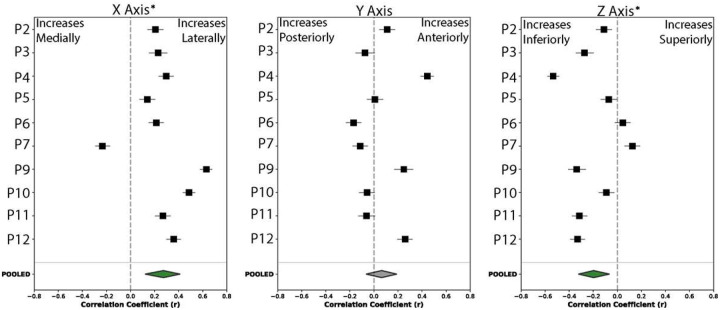
Pooled within-participant correlations between MNI-152 axis coordinates and high gamma activation strength. Forest plots show participant-level Pearson correlations (squares ± horizontal lines = 95% CI) between each electrode’s coordinate along one MNI-152 axis and its trial-averaged dB change in broadband high gamma power, computed across ten participants (P2–P7, P9–P12). Pooled estimates (diamonds, bottom) were obtained by Fisher z-transformation, averaging, and back-transformation. Green shading favors positive correlations, red shading negative correlations. **A.** Medial–lateral axis (X). Mean r = 0.273, 95% CI [0.121, 0.412]; t(9) = 3.47, uncorrected p = 0.0070, FDR-corrected p = 0.028. High gamma activity increased significantly toward the lateral hand-knob region. **B.** Anterior–posterior axis (Y). Mean r = 0.064, 95% CI [−0.065, 0.190]; t(9) = 0.97, uncorrected p = 0.357, FDR-corrected p = 0.357 (ns). **C.** Inferior–superior axis (Z). Mean r = −0.197, 95% CI [−0.323, −0.065]; t(9) = −2.91, uncorrected p = 0.0173, FDR-corrected p = 0.035. High gamma activity decreased significantly with superior displacement, consistent with the inferior tilt of the precentral gyrus as it extends laterally; the medial–lateral and inferior–superior gradients therefore partly reflect the same geometric axis (see partial correlation analyses in main text). P-values were corrected across the three anatomical axes using the Benjamini-Hochberg false discovery rate procedure. Asterisks (*) denote FDR-corrected p < 0.05; ns = not significant.

Partial correlation analyses confirmed this geometric coupling: controlling for either the medial-lateral or the inferior-superior coordinate nullified the other’s effect. Per-axis within-participant correlations across all three MNI-152 axes are shown in Extended Data Figure 6. These findings suggest that HGA recorded at this microscale may still follow observable medial-lateral organizational patterns, though the confounding of spatial coordinates and limited sample size warrant caution.

## Discussion

To our knowledge, our work provides the first comprehensive characterization of human motor cortical microstructure during movement at 400-micrometer spatial resolution — an order of magnitude finer than conventional EcoG^9,10,16–18^. The spatial extent of cortical activation appears to predict which movements can be reliably distinguished. We observed a strong correlation between high gamma magnitude and spatial extent across all regions (M1: r = 0.910, S1: r = 0.826), indicating that greater amplitude modulation is systematically associated with broader spatial recruitment. This relationship was consistent across participants with M1 coverage: participants with broader cortical engagement showed better gesture discrimination, with the moment-to-moment decoding accuracy in each of P9, P10, and P12 tracking the time course of extent of activation (P9: r = 0.711, P10: r = 0.867, P12: r = 0.778; all p < 0.001, Fig. 6A). Extension movements consistently recruited larger M1 activation areas than flexion movements, which may explain their superior decodability and lower confusion rates. We also provide the first detailed surface mapping of the entire upper extremity in human M1 and S1, revealing nested, hierarchical representations with systematic organization along the proximal-distal axis. Representational similarity analysis demonstrated clear clustering by anatomical distance, with joints actuated by forearm musculature showing greater similarity to each other than to more proximal joints. Spatial extent of activation increased systematically from individual fingers (3–6 mm^2^) through wrist and forearm to proximal joints (shoulder: 41 mm^2^, elbow: 36 mm^2^), with substantial overlap within limb segments (Dice coefficients 0.72–0.93) but progressively non-overlapping areas with anatomical distance. These findings establish both the possibilities and constraints for translating high-density cortical interfaces into functional brain-computer interfaces.

**Fig. 7.**
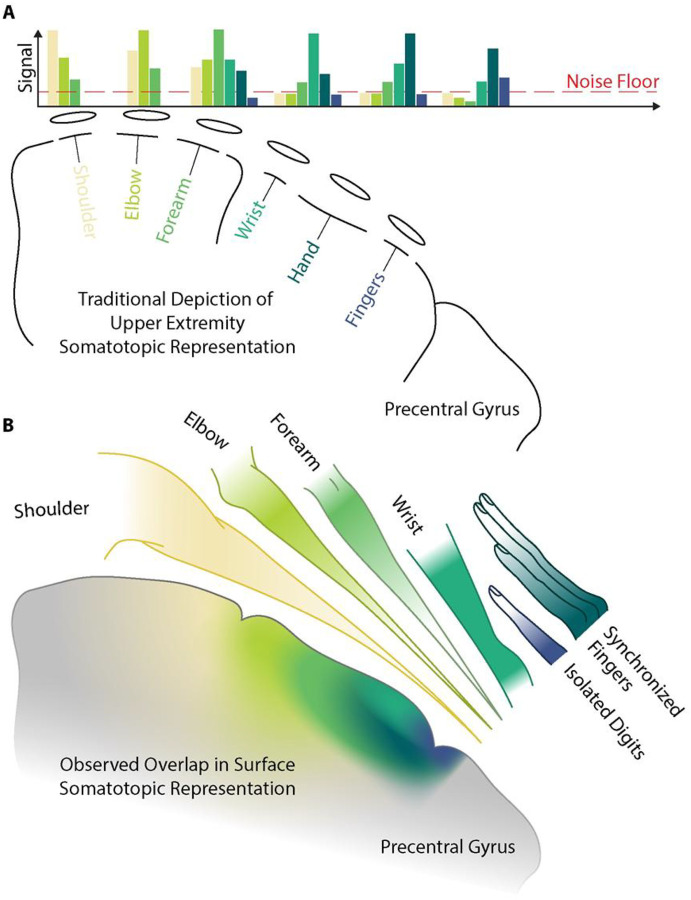
Reconciling the classical motor homunculus with a nested, overlapping model of upper-extremity surface somatotopy. **A.** Traditional “discrete” depiction of the motor homunculus in which each body effector occupies a compact, non-overlapping territory along the precentral gyrus, with larger cortical allotment to distal effectors (e.g., fingers) than to proximal effectors, shown alongside an illustrative bar diagram of the intuition behind what surface arrays can resolve: bars depict the relative amplitude of movement-evoked broadband high-gamma activity for each upper-extremity effector (shoulder, elbow, forearm, wrist, digits), and the dashed horizontal line marks the noise floor below which activity cannot be reliably distinguished from background. Only the portion of each effector’s representation that rises above the noise floor contributes to the observable footprint on the cortical surface, schematizing how a continuous, overlapping underlying organization (Panel B) can give rise to the apparently segregated, size-scaled territories of the classical homunculus. **B.** Proposed nested, overlapping model informed by the present microscale recordings. Upper-extremity representations are organized as concentrically larger cortical territories from distal to proximal effectors (Isolated Digits ⊂ Synchronized Fingers ⊂ Wrist ⊂ Forearm ⊂ Elbow ⊂ Shoulder), so that proximal-joint territories subsume the more distal representations they enclose. This arrangement is consistent with the Dice-coefficient analyses in Fig. 5, the medial-to-lateral gradients in Fig. 4 and 6, and the hierarchical clustering in Fig. 3.

Our findings on somatotopy across the surface of the brain align with fMRI literature on brain mapping that challenges the discrete homunculus model, demonstrating nested and overlapping motor representations with substantial spatial overlap between joints.^19–21^ Intracortical microstimulation studies in non-human primates similarly show interleaved forelimb representations in M1.^22,23^ Together with the hierarchical clustering in Fig. 3, the mediolateral gradients in Figs. 4 and 6, and the Dice-coefficient analyses in Fig. 5, our microscale recordings are most parsimoniously captured by a nested, overlapping model of surface somatotopy (Fig. 7), in which the cortical territory recruited by each upper-extremity effector progressively encloses the territories of more distal effectors (Isolated Digits ⊂ Synchronized Fingers ⊂ Wrist ⊂ Forearm ⊂ Elbow ⊂ Shoulder). This reframing departs from the discrete Penfield–Boldrey depiction not by reassigning effector locations but by replacing compact, non-overlapping territories with concentrically expanding ones. Proper characterization of movement-evoked activity on the surface of the brain is still limited. Most recent efforts covering representation of finger-wrist-elbow flexion/extension showed findings more localized to S1.^8^ We provide the first detailed characterization using 400-micrometer electrode spacing, revealing hierarchical organization consistent with these latest descriptions of body representation in the brain. Representational similarity analysis demonstrated systematic clustering by anatomical distance, with forearm-actuated joints showing higher similarity to each other than to proximal joints.

The observation that EoA increased systematically along the proximal-distal axis aligns with previous works in ECoG literature that showed larger effect sizes in activation for shrugging the shoulders over opening/closing the hands.^24^ To our knowledge, our work is the first to observe this relationship between magnitude of HGA change and spatial extent using surface recordings in humans.

Differences in EoA between extension and flexion of the hand align with fMRI studies showing asymmetric cortical recruitment patterns.^25^ However, we cannot fully dissociate cortical organization from biomechanical factors (e.g., potential resistance related to the motion capture glove, or extension having to overcome gravity), disease-specific effects (relative extensor weakness compared to flexion has been shown in Parkinson’s disease off medication states^26,27^), or subcortical influences (flexor versus extensor biases of subcortical and spinal cord networks may contribute to the observed asymmetry). The observed cortical predominance of extension in our data may reflect a combination of these factors rather than pure cortical physiology.

Surface potentials reflect the summed activity across millions of neurons and this observation may point to a relationship between surface local potentials and the underlying/intracortical LFP and single/multiunit activity. Our observation of a linear relationship between spatial extent and magnitude of HGA, as well as better outlining surface boundaries during movement raises the question of whether hdMEAs can isolate sources equivalent to single or multi-unit activity at depth. Alkadhi *et al*. (2002) used similar methods to characterize somatotopy in fMRI.28 This could offer immense benefit for development of BCI from these devices. This relationship could also explain the difficulty of isolating isolated finger flexion/extension representations in the surface of the brain. Decoding individual fingers has been achieved successfully post-hoc^5^ and deployed live^18^, usually following self-paced repetitions of finger movement per detection—which likely improves signal strength. Our findings suggest that complex hand gestures may be more clinically tractable than individual finger control — not due to algorithmic limitations, but because gestures inherently produce larger, more distinct cortical signatures.

The observation that representational similarity affects decoding has been described previously in papers decoding from EcoG^8,29^ Thomas *et* al. also identified that averaging activity across the full duration of the movement improved their classification accuracy by increasing difference in patterns. A two-step approach to decoding in which first the joint was decoded followed by direction was also useful in bypassing this obstacle. The former proposition however would increase latency in live implementations. Given the tradeoff between pattern similarity and decodability, strategic choice of DoFs for clinical BCIs can be used to decrease training volume and should prioritize DoFs with high impact on quality of life.^30^ A system providing reliable control of several distinct hand configurations combined with gross arm positioning may prove more functional than one attempting to decode every individual joint at reliability’s cost.

The importance of electrode placement is also a recurring topic in ECoG literature, especially in the use of mEA. These types of electrodes have the added benefit of having higher SNR which allows for better discernability between broadband HGA and the noise floor^31^. However, this comes with the caveat that they record from a smaller radius (although the extent of this radius is still a matter of investigation). To our knowledge, this is the first demonstration of spatial gradients across participants from measurements at the microscale. If this relationship persists with sufficient number of participants, these findings can provide actionable guidance on placement of non-penetrating BCI on the surface of the brain. These gradients rely on projecting brain for each participant into shared spaces. Projecting individual participant brain surfaces into coordinate systems that better preserve the relationship around the central sulcus has been shown to be beneficial in a clinical application for neuromodulation^32^ and may be beneficial in the future if the priority is to plan deployment of motor BCI. The “fsaverage” brain’s rendering of a proposed normative surface of the whole brain may come at the expense of distorting features important for ECoG placement.

In our patient cohort, we evaluated decoding accuracies across four different gyri. In P10, channels in both M1 and S1 decoded above chance. Participant 6, with electrodes in M1 and in the junction between PCG and SFG did not achieve above-chance decoding despite showing channels with significant HGA modulation during hand movement. In recent neurophysiological studies, observation of non-specific changes in the motor cortex has been described in the context of motor association areas (at depth, in sEEG studies^11^) and inter-effector regions (in fMRI^19^), which might explain this finding. Decoding from any of those regions might be reasonable from the context of BCI. The observed HGA in S1 for P10 likely reflects proprioception.^29,33^ Some patients with spinal cord injury show residual proprioceptive function and may benefit from this. Furthermore, encoding proprioceptive feedback into BCIs has been shown to improve decoding^34^ and is an active field of investigation.

Of note, the observed spatiotemporal profile observed over MFG for P4 shows potential for BCI applications. Decoding accuracy was comparable to that achieved for M1 electrodes for P9, P10 and P12. The fact that those electrodes showed specificity for hand movement might reflect specific surface somatotopy for that gyrus or potentially learning-related effects. The lack of UE representation aside from the hand may prove useful in decoding dexterous hand movements at the expense of losing other DoF from the UE.

Limitations of our study include the limited number of trials available for the isolated joint movements. As such, we could not test decoding accuracy for these movements, and any generalization of our findings on representational similarity and overlap to decodability for upper extremities should be validated in future experiments. We chose logistic regression for its decoding interpretability; however, as with any model, the choice of architecture can confound conclusions drawn about the relationship between decoding and HGA, though the patterns we describe agree with previous studies. This choice was made so model architecture did not take precedence over interpretability.

A key limitation is the variability in optimal array placement across participants. While cross-participant analysis revealed consistent spatial gradients, arrays were positioned across multiple gyri (M1, S1, MFG, SFG) due to individual neuroanatomical differences and surgical constraints. The hand knob and central sulcus provide reliable reference points for targeting hand representations, but larger cohorts are needed to validate whether the organizational principles we observed generalize across the broader population and determine optimal placement strategies for clinical deployment.

Future work should address decoder architectures that can exploit hdMEA spatial patterns while managing the computational burden of high channel counts and sampling rates. Dimensionality reduction techniques that leverage spatiotemporal patterns, such as convolutional neural networks or spatial filtering approaches, may capture the nested, overlapping representations we observed more efficiently than treating each channel independently. Such methods could reduce computational costs and overfitting risks while remaining robust to the representational overlap inherent in motor cortex organization, enabling more practical real-time BCI deployment.

Our work establishes that high-density microelectrode arrays can resolve motor cortical representations at unprecedented spatial scales, revealing organizational principles that constrain and inform BCI decoder design. Translating these insights into clinical benefit requires navigating the challenges associated with reliably interpreting micrometer-scale local physiology.

## Methods

### Patient Enrolment and Consent Process.

This study was approved by the Institutional Review Board of the University of Pennsylvania under protocol number #852828. Patients of the movement disorders clinic of the Pennsylvania Hospital were considered fit for inclusion if they were undergoing unilateral or bilateral awake DBS implantation for treatment of Parkinson’s disease or essential tremor. Invitation to participate was extended regardless of gender. Consent to participate in the study was granted one to two weeks before the scheduled surgery and then again on the morning of the surgery. Patients were not compensated for participation in this study.

### hdMEA Device.

Cortical neural data was recorded using the Layer 7 array from Precision Neuroscience^31^. The Layer 7 hdMEA array is made of a thin (22 micrometers), flexible polyimide substrate and includes 1024 platinum recording contacts over a 1.2 × 1.2 cm area. The contacts themselves come in three types: There are 5 reference (mega) channels with a diameter of 500 micrometers located on the edges of the array, 42 macro contacts interspersed with a diameter of 380 micrometers, and 977 micro contacts interspersed with a diameter of 50 micrometers. The intercontact distance (center to center) between all micro and macro contacts was 400 micrometers. The arrays are bonded to custom recording boards with high-density amplifier chips (Intan Technologies) and housed in a hardshell case for protection during handling. Serial peripheral interface cables connect the recording boards to the data acquisition system for recording and real-time data visualization and processing. The array, recording boards, housing, and cables are all sterilized prior to surgery using ethylene oxide (EtO).

For spatial analyses requiring a geometric grid representation, including Representational Similarity Analysis (RSA) (see Representational Similarity and Overlap Analysis section), our spatial mapping function maps 4 of the 5 reference electrodes to the 4 nearest corner positions of the 33×31 electrode grid, allowing these electrodes to be incorporated into the spatial framework. One reference electrode could not be appropriately mapped and was excluded. This spatial mapping procedure resulted in 1023-dimensional feature vectors (1,019 grid channels + 4 closest reference electrodes) for RSA and other grid-based analyses. Non-spatial analyses (e.g., trial-averaged power calculations) used the full 1024 recording channels without the spatially-mapped references.

### Preoperative Planning and BCI Implantation.

In each case, the patient was brought to the operating room (OR) and set up for a standard-of-care awake DBS surgery. A stereotactic frame (Leksell Vantage Frame, Elekta) was placed while the patient was under light sedation. An intraoperative imaging platform (O-arm, Medtronic) was then placed around the patient’s head and a fiducial box placed on the frame to define the stereotactic coordinate system and to subsequently allow for intraoperative confirmation of microelectrode cannula location. Burr holes were planned based on the desired subcortical target for the patient’s movement disorder and the number of electrodes to be implanted for clinical purposes. After the burr hole was made, the dura was opened and a custom surgical guide was used to gently slide the hdMEA array towards the targeted location, similar to previously published stereotactic techniques for placement of low-density ECoG strips during DBS procedures^35,36^. hdMEA targets the hand knob — a distinct Ω-shaped region within the precentral gyrus of the primary motor cortex (M1), measures approximately 20–30 mm in length, 8–15 mm in width, and 2–4 mm in cortical thickness, playing a critical role in fine hand and finger movement control.^37^

After hdMEA placement, the microelectrode cannula was placed using the stereotactic frame to the desired subcortical target. We performed the intraoperative O-arm spin after microelectrode insertion to post-operatively reconstruct the microelectrode position relative to the intended subcortical target and to localize the hdMEA. A custom hdMEA reconstruction algorithm has been developed that leverages three radio-opaque fiducial markers fabricated on the hdMEA. These markers can be identified as high-intensity voxels on the intraoperative CT. By merging the pre-operative stereotactic protocol MRI with the intraoperative CT, we used the marker-relative electrode positions to localize each channel on the participant’s reconstructed native brain anatomy. To allow for inter-subject comparison, each channel’s coordinates were then mapped to both fsaverage (MNI305) and MNI152 spaces. Array reconstruction, projection, and mapping were carried out in RAVE^14^ and YAEL.^15^

### Kinematic Data Acquisition and Processing.

Participants were fitted with motion capture gloves (Stretchsense MoCap Pro) and an inertial measurement unit system (Movella Xsens Awinda) to capture hand gestures before patients were moved to the operating room. The Xsens system comprises inertial sensors that measure position relative to a reference sensor placed on the lower back, recording each sensor’s location in a Cartesian coordinate system. Eleven sensors were positioned on the upper body: bilaterally on the shoulder, upper arm, forearm, and hand, plus additional sensors on the head, sternum, and lumbar region. The Stretchsense glove uses capacitive sensors to detect changes in finger position. The native Xsens software automatically integrates the 20 glove sensor measurements into the common coordinate frame established by the body sensors.

To detect movement onsets, we computed the first derivative of positional measurements for the index and pinky fingers across x, y, and z axes. The Euclidean norm of these velocity components was calculated as the square root of the sum of squared values. Movement onset detection was performed by identifying the first velocity peak following the visual cue, then tracing backward and forward to an empirically-determined velocity threshold to define movement start and end times. All automatically detected onsets and offsets were manually verified and corrected for each participant prior to any analyses. Any movement trials that were not prompted by a visual cue (see “isolated joint paradigm” below) had to be manually segmented based on velocity curves following a similar process and evaluating velocity curves for the relevant sensors.

For each trial, kinematic data spanning from 2 seconds before to 2 seconds after movement onset were extracted. Velocity magnitudes were computed for hand and wrist sensors using central differences of position data across x, y, and z coordinates. Trials were excluded from physiological analyses based on two velocity-based criteria using pooled statistics across all recordings: (1) hand velocity during baseline periods (before −250 ms or after 750 ms relative to movement onset) exceeded the mean plus 4 standard deviations, indicating extraneous hand movement during rest; or (2) the magnitude of hand-wrist velocity difference during the movement period (−250 ms to 750 ms) exceeded the mean plus 4 standard deviations (see **Supplemental Fig. 7**), indicating excessive arm movement, which might confound hand activation profiles. The 4 standard deviation threshold was chosen to provide conservative trial exclusion while accounting for multiple comparisons across time points. These excluded trials were retained for decoding analyses.

### Rock-paper-scissors paradigm.

After patients surface from anesthesia, microelectrode recordings of the subcortical target are performed as part of standard clinical care to establish the borders of the subcortical target. Once the subcortical structure has been delineated, approximately 20–25 minutes are dedicated to the intraoperative experiment.

Each trial begins with an audio cue. A visual cue of the target gesture appears 1–1.5 seconds later (with the delay drawn from a uniform random distribution). The patient imitates the gesture shown. A member of the experimental team observes the gesture, confirms the gesture is correct, and delivers a success or fail prompt that is displayed for 1 second. The next trial starts immediately after the prompt, beginning with the audio cue.

The patient performs pre-determined sequences of gestures prompted by a visual cue displayed on a monitor screen. Gesture nomenclature was as follows: rock — all fingers fully flexed to close the hand, occluding the ventral surface of the hand; paper — all fingers fully extended showing the palm of the hand; scissors — all fingers fully flexed while the second and third digits are held in full extension. Also referred to colloquially when signing the number 2, or commonly referred to as a “peace” sign. Datasets were composed of recordings of variable durations ranging from 20 to 60 trials each (Extended Data Table 1). The gesture sequences followed a repetitive set of gestures (fixed-sequence) or a balanced random sequence of gestures (random-sequence). For each patient, a single recording was done under the fixed-sequence paradigm, and all other recordings were collected using a random-sequence. Gesture sequences for participants 4 and 6 included transitions between all gestures, while gesture sequences for participants 7–12 always started and returned to the baseline gesture “Rock.” For example: Rock, Paper, Rock, Scissors, Rock. The transitions between the scissor and paper gestures in participant 4 and 6 dataset were therefore excluded from analysis. Transitions between rock and paper or scissors were considered extension trials. The opposite was considered flexural. Balanced randomization was used to ensure a constant number of trials of paper and scissors. A random sequence of gestures used a random choice between “scissors” and “paper” from the starting “rock” transition at any given point. The differences between neural representations between fixed-sequence and random-sequence paradigms were not explored in this paper.

### Isolated joint paradigm.

Participants were guided by a member of the experimental team to follow along to isolated movements of specific joints. These were carried out from a lateral to medial fingers, and then from distal to proximal joints along the arm.

Participants followed along at their own pace for a duration between 5 and 10 seconds. The recruited joints were individual fingers (flexion/extension), wrist (flexion/extension), forearm (pronation/supination), elbow (flexion/extension), shoulder (abduction/adduction). This recording was left for last and had varying lengths depending on the amount of allotted research time left for any given case. As a result, a varying number of movements, and interval between movement categories, were observed, and no predetermined baseline period was established. Baseline periods were defined by segments without any movement detected across all sensors of the body for any given participant (**Supplemental Fig. 2**).

### Signal Processing.

Impedance was measured for each electrode following burr hole cover placement. Electrodes with an impedance measurement above 2 MΩ were excluded from analysis. Neural data was common average referenced across low-impedance electrodes by subtracting the mean voltage of all low impedance electrodes from the voltage at each electrode at every time point in the recording. Subsequently, high impedance channels were imputed with zeroes for multitaper spectral estimates.^38^ The multitaper method applies a set of discrete prolate spheroidal sequences (DPSS) as orthogonal tapers to the data. The Fourier transform of each tapered segment is computed, and the resulting spectra are averaged. This approach reduces spectral leakage and variance compared to single-taper methods, decreasing the variance in frequency estimation.^38^ Time-frequency estimates were conducted for sliding time windows of 100 milliseconds each at 90% overlap, and a frequency resolution of 40 Hz. The relationship between the time-bandwidth product (*NW*) and the maximum number of tapers (*T*) to be used follows a relationship of

T=2*NW−1

in which NW can be calculated by multiplying the size of the time window in seconds by the ½*frequency resolution (*F*).^39^ The broadband high gamma power was obtained by averaging across frequency bands in the interval between 80 and 200 Hz.

### Quantifying event-related potentials and calculating activity effect size.

Event-related potentials were calculated by segmenting neural data into epochs of ±2000 ms centered on verified movement onset times. The power spectral density was first subjected to frequency normalization to correct for the characteristic 1/f spectral decay inherent in neural recordings. This was done by dividing the power at each frequency by its average across the entire recording to yield frequency-corrected values. Following this normalization, high gamma band activity was isolated by selecting frequencies between 80–200 Hz and averaging power across this frequency range. A baseline period was defined as the 500 ms preceding movement onset, during which participants maintained a resting hand posture.

To quantify the magnitude of neural modulation associated with movement, we calculated decibel (dB) changes from baseline using the formula 10×log_10_(trial_power / baseline_power). However, to account for cross-trial variability that dB changes do not capture, we additionally computed a signed coefficient of determination (signed r^2^) as the primary effect size metric for each electrode. This approach has been used extensively in neuroscience literature with different penalization terms for sample size imbalances. The version we used was the one published most recently.^11,12^ The signed r^2^ quantifies the percentage of variance in spectral power that corresponds to a given motor condition when compared against rest. This metric was calculated using the formula:

$$r_{mr}^{2}=\left(\frac{{\left(\bar{m}-\bar{r}\right)}^{3}}{|\bar{m}-\bar{r}|\sigma_{mur}^{2}}\right)\left(\frac{N_{m}N_{r}}{N_{mur}^{2}}\right)$$

where $\bar{m}$ and $\bar{r}$ represent the mean power during movement and rest conditions respectively, σ^2^_m_∪_r_ is the pooled variance across both conditions, and N_m_ and N_r_ are the number of trials in each condition. The sign of r^2^ was preserved to indicate the direction of modulation, with positive values reflecting power increases and negative values reflecting power decreases relative to baseline. This signed r^2^ measure provides a more complete characterization of neural modulation than traditional power change metrics alone, as it accounts for the consistency of the effect across trials.

For the isolated joint paradigm, periods of rest with duration of 500 ms were chosen where magnitude of the velocity was observed to be below the 1st percentile across the full recording. Segments were chosen if they were 1 or more seconds away from any movement and 500 or more milliseconds from any other baseline periods. This pool of rest trials was used to compute the baseline distribution for the dB and signed r^2^ analysis.

### Statistical Testing and correcting for family-wise error rates.

Statistical significance was assessed using a non-parametric permutation test that controls the family-wise error rate across all electrodes.^40^ To build a null distribution, we performed 5,000 permutation iterations. In each iteration, trial labels were randomly shuffled between movement and rest conditions, pooling all trials regardless of their original condition assignment. For each shuffled dataset, we computed a new t-statistic map and then calculated Threshold-Free Cluster Enhancement (TFCE) scores^13^ separately for both positive and negative t-values. The maximum TFCE score across all electrodes was recorded for each direction independently. This procedure generated null distributions of the maximum TFCE statistic under the assumption of no true difference between conditions. For each electrode, the observed TFCE score was compared against the appropriate directional null distribution. The p-value was calculated as the proportion of permutation maxima that equaled or exceeded the observed value, adjusted by adding one for the observed data itself and dividing by the total number of permutations plus one. Electrodes were deemed statistically significant if their p-value fell below *α* = 0.025 for either the positive or negative direction (yielding a two-tailed familywise *α* = 0.05).

### TFCE Score calculation.

The TFCE score for each electrode was calculated by integrating over threshold heights (*h*) with a step size (*dh*) of 0.1:

$$\text{TFCE}=\sum h^{H}\times {\text{extent}}^{E}\times dh$$

where extent is the number of spatially contiguous electrodes exceeding threshold *h*, and *H* = 2.0 and *E* = 1.0 are parameters that weight the influence of signal height and spatial extent, respectively.

This approach was chosen to circumvent the inferential fallacies that can arise from methods such as cluster-based permutation testing, which require predetermined cluster-defining thresholds and can produce clusters with arbitrary spatial characteristics.^41^ The parameter values (H=2.0, E=1.0) were selected based on prior work that showed the effect of these parameters on datasets of varying signal-to-noise ratios.^42^

### Quantifying Extent of Activation.

To quantify the spatial extent of neural modulation for each movement, we computed the extent of activation (EoA) based on statistically significant channels identified by TFCE analysis (see Statistical Analysis section). Only channels showing both statistical significance (p < 0.05, two-tailed, TFCE-corrected) and positive modulation (signed R^2^ > 0) were included in area calculations. This metric captures both the strength of individual electrode responses (magnitude of r^2^) and the spatial extent of activation (number of significant electrodes), providing a unified measure of cortical engagement during movement.

Spatial extent was also calculated for spatial similarity analyses. Each electrode in the array was modeled as having a circular footprint with a radius of 200 micrometers (half the 400 micrometers electrode pitch), corresponding to an area of π × (0.2 mm) ^2^ ≈ 0.126 mm^2^ per electrode. The total spatial extent for each movement was calculated as the sum of individual electrode areas across all significantly modulated channels.

### Correlation Analysis and Multiple Comparison Correction.

Relationships between continuous variables (e.g., HGA magnitude and spatial extent, decoding accuracy, and extent of activation) were assessed using Pearson product-moment correlation coefficients.

Confidence intervals for correlation coefficients were calculated using Fisher’s z-transformation. For within-participant correlations, individual r values were Fisher z-transformed, averaged, and back-transformed to obtain pooled effect sizes with 95% confidence intervals. For analyses involving multiple statistical tests, we applied false discovery rate (FDR) correction using the Benjamini-Hochberg procedure. Specifically, spatial gradient analyses testing correlations across three anatomical axes (medial-lateral, inferior-superior, anterior-posterior) were FDR-corrected (reported in Spatial Gradients section). Individual correlations testing specific a priori hypotheses (e.g., relationship between extent of activation and decoding performance) were not corrected for multiple comparisons, following standard practice for hypothesis-driven analyses. Statistical significance was set at α = 0.05 for all tests unless otherwise specified.

### Representational Similarity and Overlap Analysis.

To assess the spatial similarity between movement representations, we computed pairwise overlap using the Dice coefficient. For each pair of movements, binary activation masks were created from the statistically significant positive channels and mapped to the 33×31 spatial grid corresponding to the array geometry. The Dice coefficient was then computed as:

$$\text{DICE}=\frac{2\times |A\cap B|}{\left|A|+|B\right|}$$

where A and B represent the sets of significantly activated channels for the two movements, |A ∩ B| denotes the number of channels activated by both movements (intersection), and |A| and |B| denote the total number of channels activated by each movement. The Dice coefficient ranges from 0 (no overlap) to 1 (complete overlap), providing a normalized measure of spatial similarity that accounts for differences in the total extent of activation between movements.

### Representational Similarity Analysis.

To characterize the similarity of neural activity patterns across movements beyond spatial overlap alone, we performed representational similarity analysis (RSA) using the continuous-valued high gamma power modulation across all electrodes.^43^ Spatial grids for dB changes from baselines were flattened into a 1,023-dimensional feature vector for each movement (1 reference electrode was excluded as it was more distant from the remaining).

We computed pairwise dissimilarities between all movement pairs using Euclidean distance, constructing a representational dissimilarity matrix (RDM). For movements i and j with activation vectors *x_i* and *x_j*:

$$d(i,j)=||x_{i}-x_{j}|{|}_{2}$$

The resulting RDM captures the overall pattern similarity across the entire electrode array, with smaller values indicating more similar neural representations. The RDM approach considers the full continuous distribution of activation magnitudes across all electrodes, providing a more complete measure of representational similarity than simply spatial overlap.

To visualize the high-dimensional representational space, we applied multidimensional scaling (MDS) to the dissimilarity matrices.^44^ MDS projects the high-dimensional activation patterns into 2D and 3D spaces while preserving pairwise dissimilarities as closely as possible. The quality of these low-dimensional embeddings was assessed using Kruskal’s normalized stress:

$$\text{Stress}=\sqrt{\frac{\sum {\left(d_{\text{original}}-d_{\text{projected}}\right)}^{2}}{\sum {\left(d_{\text{original}}\right)}^{2}}}$$

where *d_original* represents the original dissimilarities from the RDM and *d_projected* represents distances in the MDS embedding space. Lower stress values indicate better preservation of the original dissimilarity structure, with values below 0.10 generally considered good fit.^45^

To identify hierarchical structure in movement representations, we performed agglomerative hierarchical clustering on the dissimilarity matrices using ward linkage method. This analysis reveals nested groupings of movements with similar neural representations, which can be visualized via dendrograms with branch heights reflecting the dissimilarity at which clusters merge.

### Comparing Flexion and Extension Movement Components.

To assess differences in cortical activation between flexion and extension movements, we analyzed trial-level high gamma power averaged across the movement execution period. For each trial, the averaging window was defined from the time of first statistically significant neural modulation (lags determined from TFCE analysis of pooled data) to the end of the movement as detected by kinematic sensors. This window was chosen to capture both preparatory and execution-related neural activity while excluding periods without meaningful task-related modulation.

Within this window, high gamma power was first averaged temporally for each electrode, then averaged across all electrodes to yield a single value per trial. Trials were classified as flexion or extension based on the gesture transition paradigm: transitions from Rock (closed hand) to Paper or Scissors were classified as predominantly extension movements, while the reverse transitions were classified as predominantly flexion movements. Statistical comparisons between flexion and extension trials were performed using independent samples t-tests for each gesture pair (e.g., rock-to-paper vs paper-to-rock).

### Multivariate pattern analysis, feature extraction and temporal windowing.

To assess the temporal dynamics of gesture representation in the M1, we performed multivariate pattern analysis (MVPA) using a temporal generalization framework.^46^ Neural activity from all electrodes was used as input features, with no dimensionality reduction applied to preserve the full spatial information content of the array.

For each trial, high gamma activity was extracted using causal temporal windows, where each timepoint represented the end of a 100 ms integration window (10 samples at 10 ms resolution). This causal approach ensures that decoder predictions at any given timepoint reflect only information available up to and including that moment, preventing information leakage from future timepoints. Temporal windows were extracted at 50 ms intervals spanning from 1000 ms before to 1000 ms after movement onset, yielding a time-resolved representation of neural activity throughout the movement period. For each window, the neural data were flattened into feature vectors of dimensionality (number of channels × window length), preserving both spatial and short-timescale temporal structure.

### Multivariate pattern analysis, classification framework.

We trained separate logistic regression classifiers for two labeling schemes: (1) gesture identity, classifying all four hand transitions (rock-to-paper, rock-to-scissors, paper-to-rock, scissors-to-rock), and (2) finger-specific movement, classifying index finger states (flexion, extension, active hold) independent of pinky position. All classifiers used L1 regularization (*C* = 0.1, where *C* is the inverse regularization strength) to promote sparse solutions and reduce overfitting given the high-dimensional feature space. For multi-class problems, we employed a one-versus-rest strategy, training independent binary classifiers for each class and selecting the class with the highest decision function value at test time.

### Multivariate pattern analysis, dataset balancing.

To address class imbalance in the gesture dataset, we applied class-specific undersampling when the ratio between the most and least frequent classes exceeded 1.2. For each labeling scheme requiring balancing, trials were randomly subsampled without replacement to match the size of the smallest class, with balanced datasets created independently for each classification problem to avoid artificially constraining the available data.

### Multivariate pattern analysis, temporal generalization analysis.

Model performance was evaluated using a temporal generalization framework, in which classifiers trained at each timepoint were tested both at the same timepoint (diagonal decoding) and at all other timepoints (cross-temporal generalization). This approach reveals both when information becomes decodable and how stable neural representations are across time. All analyses used 5-fold stratified cross-validation to ensure balanced class representation in each fold and to obtain robust estimates of generalization performance. For each train-test timepoint pair, we computed both classification accuracy and area under the receiver operating characteristic curve (AUC). For binary classifications, AUC was calculated using the predicted probability of the positive class. For multi-class problems, we used the one-versus-rest macro-averaged AUC, which treats each class as the positive class in turn and averages the resulting binary AUC scores.

The temporal generalization matrix was constructed by training independent classifiers at each timepoint and evaluating them across all timepoints, yielding an *n_train × n_test matrix* where each element *(i,j)* represents the performance of a classifier trained at timepoint i when tested on data from timepoint *j*. High values along the diagonal indicate successful decoding at specific time windows, while extended off-diagonal patterns reveal temporal stability of neural representations. Decoding performance was evaluated relative to chance level, defined as 0.50 for binary classification and 1 over the number of classes for multi-class problems.

### Correlation to effect size metrics.

To relate encoding strength to decoding performance, we computed Pearson correlations between time-resolved decoding accuracy (diagonal AUC from MVPA) and summed signed r^2^ values derived from the encoding analysis. Signed r^2^ values were summed across TFCE-significant electrodes within each temporal slice to yield a scalar encoding strength metric, preserving effect direction. Encoding time courses were interpolated to the decoding time axis using cubic splines, with values outside the observed encoding range set to zero. Correlations were computed separately for each participant and cortical region.

### **Spatial Gradient Analysis**.

To assess whether neural encoding strength varied systematically along anatomical axes, we computed within-participant Pearson correlations between each electrode’s MNI-152 coordinates (x: medial-lateral, y: anterior-posterior, z: inferior-superior) and trial-averaged HGA (dB). For each participant and axis, correlation coefficients were calculated across all electrodes with valid recordings. To obtain pooled effect sizes across participants, individual correlation coefficients were Fisher z-transformed, averaged, and back-transformed to yield mean r values with 95% confidence intervals. Statistical significance of pooled correlations was assessed using one-sample t-tests on Fisher z-values against a null hypothesis of r = 0. Because three anatomical axes were tested, p-values were corrected for multiple comparisons using the Benjamini-Hochberg false discovery rate (FDR) procedure to control the expected proportion of false positives at α = 0.05.

## Extended Data

**Extended Data Table 1.** Individual participant characteristics, as well as those for the therapeutic intervention and the recordings utilized to derive their data.

## Supplementary Material

This is a list of supplementary files associated with this preprint. Click to download.

- extendeddatatable1.docx
- supplementalfinal.docx

## Data Availability

Analysis code used to generate the results reported here will be made available upon request. Trial-level broadband high-gamma recordings for the participants included in the analyses, as well as de-identified raw neural data, are available from the corresponding author upon reasonable request.
